# Protocol for a feasibility evaluation of a Social and Emotional Learning (SEL) programme to improve resilience and academic achievement in refugee children from a community learning centre in Malaysia: PARSEL (Participatory Action Research on SEL)

**DOI:** 10.1371/journal.pone.0273239

**Published:** 2022-08-18

**Authors:** Kwong Hsia Yap, Ashley Koh, Anesha Kumar, Magdalene Lahpai, Kah Hoe Cheng, Tharsini Ravindaran, Priya Vasu, Sharuna Verghis

**Affiliations:** 1 Jeffrey Cheah School of Medical and Health Sciences, Monash University Malaysia, Selangor, Malaysia; 2 Health Equity Initiatives, Kuala Lumpur, Malaysia; Public Library of Science, UNITED STATES

## Abstract

This paper describes a protocol for the feasibility evaluation of the Participatory Action Research on Social and Emotional Learning (PARSEL) programme. PARSEL aims to contribute towards the development of academic achievement and resilience among urban refugee students in a community learning centre in an upper middle-income country. The evaluation is a single arm pre-post design using a mixed methods approach, with the main focus on the feasibility of the programme. Measurements of impact are also included as the secondary outcomes of the study. The programme aims to enrol students from refugee background in a community learning centre. The programme is estimated to run for 18 months and the study is due to report in the end of fourth quarter of 2022.

## 1. Introduction—Background and rational

### 1.1 Refugees in malaysia

As of end March 2021, there were some 178 920 refugees and asylum-seekers registered with the United Nations High Commissioner for Refugees (UNHCR) in Malaysia of whom some 68% are men, while 32% are women [1]. Children under the age of 18 comprise about 26% of the displaced population in the country [1]. The protection environment for refugees and asylum seekers in Malaysia remains weak and punitive. The country is neither a signatory to the 1951 Convention Relating to the Status of Refugees, nor its 1967 Protocol. Furthermore, Malaysia has not enacted legislation recognizing the legal status of asylum seekers, refugees and stateless persons. Refugees and asylum seekers are still considered “illegal immigrants” under the Immigration Act 1959/63 (Act 155).

Malaysia provides a 50% discount off the foreigner’s rate for medical fees incurred by UNHCR recognized refugees and asylum seekers and issues birth certificates to children of refugees who are born in Malaysia. However, refugee children are disallowed from joining state run schools. Enrolling in state run schools require official documents that are state recognised. Private educational institutions are inaccessible to refugees because of the exorbitant cost of private education, and the lack of official documents and legal residence permit. This has led refugees to enrol in Community Learning Centres (CLCs) established by refugee communities, and individuals or non-governmental organisations (NGOs), with the support of UNHCR. The requirement for official documents extends to the registration for state recognised grade level examinations. This means that even though refugee children are able to attend CLCs, they are unable to register and take examinations conducted by the government that provide certifications to advance them to higher education.

### 1.2 The challenges faced by Community Learning Centres (CLCs)

Among the major challenges encountered by CLCs are lack of certification and access to public examinations, high turnover of teachers and minimal compensation, security and safety issues faced by the students and teachers in and out of school, and lack of data on out of school children. Most CLCs are severely underfunded, rely on UNHCR for teacher compensation, are overcrowded, lack proper classrooms, do not have sufficiently trained teachers, and students often lack exposure to sports or other recreational activities integral for childhood development [2]. Many CLCs use the Malaysian national syllabus; yet, there is no formal certification of learning by any authority because the lack of state recognised documents deters refugee students from sitting for examinations and obtaining education level certifications. Additionally, CLCs are considered ‘irregular’ as they are unable to obtain official registration as educational institutions. As such, CLS are subject to closure at any time by the authorities, bringing about an instability for the CLCs and continued education for the children.

Published literature reveals that a major problem in refugee schools in Malaysia was high absenteeism and dropping out of school, besides bullying, and lack of cooperation between school staff and parents among others [3]. The above findings concur with previous findings of UNICEF which also attributes the high absenteeism and dropping out of school by refugee children to the pressure to work and support families, especially for adolescents, the inability of families to afford education, and living in remote areas [2]. Another study conducted in a refugee school revealed that the students in the study experienced socio-emotional difficulties and their teachers experienced stress [4], while yet another study on sexual health knowledge with school age Chin and Kachin children revealed low levels of knowledge about their body and safe sex [5]. In the latter study, almost a fifth of the children, mostly boys, reported inappropriate sexual contact. About three out of four children stated that they did not know where to go for help if someone forced physical intimacy on them [5].

### 1.3 The rationale for Social and Emotional Learning (SEL) for CLCs

Social and emotional learning (SEL) is the capacity to recognize and manage emotions, solve problems effectively, and establish positive relationships with others [6]. Research shows that SEL promotes health enhancing behaviours and has positive effects on academic performance, benefits physical health, and reduces the risk of maladjustment, failed relationships, interpersonal violence, substance abuse, and unhappiness [6].

Within the refugee school context, the rationale for such a strengths-based intervention is further augmented by a growing body of evidence that demonstrates that schools play a pivotal role in the behavioural and social adaptation of refugee children [7,8] by undertaking educational, inter-cultural and therapeutic activities [9,10], and even promoting their development as competent adults and inhibiting the development of long term psychological sequelae when there has been the experience of childhood traumatic events [10]. This is made possible through the unique strength of schools to provide safety and support to refugee children and their parents in non-stigmatizing ways [11], and foster social connectedness for the children and their parents with the wider community [10].

SEL offers promising results for refugee children through evidence demonstrated by several SEL interventions undertaken with refugee youth which have shown positive effects [12]. While there is existing evidence that showed the positive effects of SEL in immigrant/refugee children in developed countries, the same cannot be said for SEL in disadvantaged populations in low resource developing countries. This protocol aims to evaluate the feasibility of a SEL programme that will be implemented in a CLC in Malaysia.

## 2. Methods

### 2.1 Aims and objectives

The aim of the study is to evaluate the feasibility of a complex intervention on social and emotional learning, focused on increasing resilience and academic performance in refugee children in the context of a community learning centre. The study also aims to evaluate the effects of the SEL intervention on students and teachers in the CLC.

Our primary research question is:

- Is PARSEL feasible in the context of CLC in terms of acceptability and implementation, from the perspective of teachers?

Our secondary research questions are:

- Does PARSEL have any effects in improving resilience and academic performance in refugee students?
- Do any of the proposed mechanisms in the conceptual framework explain the link between the programme, resilience and academic achievement? (Was the theory of change realised in the study design?)

### 2.2 Ethics

The study has been reviewed and has obtained ethics approval from the Monash University Human Research Committee (MUHREC Project ID 22971). Written informed consent will be obtained from all participants involved in this study.

### 2.3 Conceptual framework

**Fig 1:**
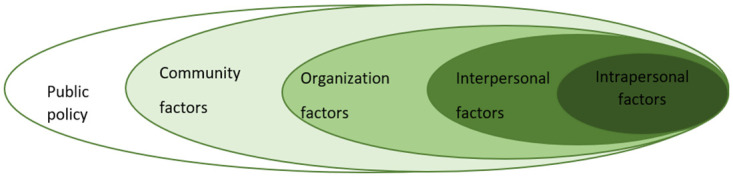
Social ecological Model (modified from McLeroy et al, 1988 [13]).

The programme is intended to help the school build and sustain a culture of SEL by implementing a multi-pronged SEL intervention using a participatory action research approach. The conceptual framework for this evaluation is based on McLeroy et al’s social ecological approach for health promotion [13]. McLeroy’s ecological model targeted both individual and social environmental factors in health promotion interventions (Fig 1). It emphasised the importance of interventions directed at changing intrapersonal, interpersonal, organizational, community, and public policy factors which support and maintain unhealthy behaviours. According to this model, changes in individuals can be produced by appropriate changes in the social environment. The implementation of environmental changes needs to be supported by the individuals in the population to produce the desired outcomes.

There is a large body of evidence on the effectiveness of school-based SEL interventions in fostering social-emotional skills (for example in emotions management, positive goal setting and achievement, empathy, maintaining and establishing positive relationships and in making responsible decisions) in students [14], that can lead to an improvement in a wide range of student outcomes, including positive social and emotional development, mental health, and academic attainment [15,16]. The improvement in academic performance was linked to the reduction of negative behaviours, ability to manage stressors and better attitudes about themselves which then would result in greater attachment, engagement and commitment to lessons and school [15,17,18]. SEL is also linked to resilience [19], as SEL enhances positive behaviours and factors which also promote resilience. To be resilient is to successfully adapt despite difficult/stressful circumstances [20,21]. Resilience is dependent on multiple systems of influence in which the risk and protective factors are found within individuals and also within the levels of environment individuals are in [22]. SEL interventions have shown effectiveness in creating supportive learning environments and positive student-teacher relationships which are also protective factors of resilience [23]. The internal and external environment resulting from successful SEL interventions are generally aligned with conditions that contribute towards resiliency promotion and improved academic performance.

Research has shown that the success of SEL interventions in schools can be affected by the factors related to implementation [15]. Teachers are the key drivers of SEL programmes and practices in schools. Their own well-being, resilience and social-emotional competence have strong influence on their students and are factors that determine the extent and success of SEL implementation in schools [24–26]. Teachers are therefore crucial in the provision of a supportive learning environment in which SEL skill development and practice can occur [26].

In affecting changes within the factors found in the levels of individual and social environment of students, the programme aims to develop resilience and improve the academic performance of refugee students in the CLC.

**Fig 2:**
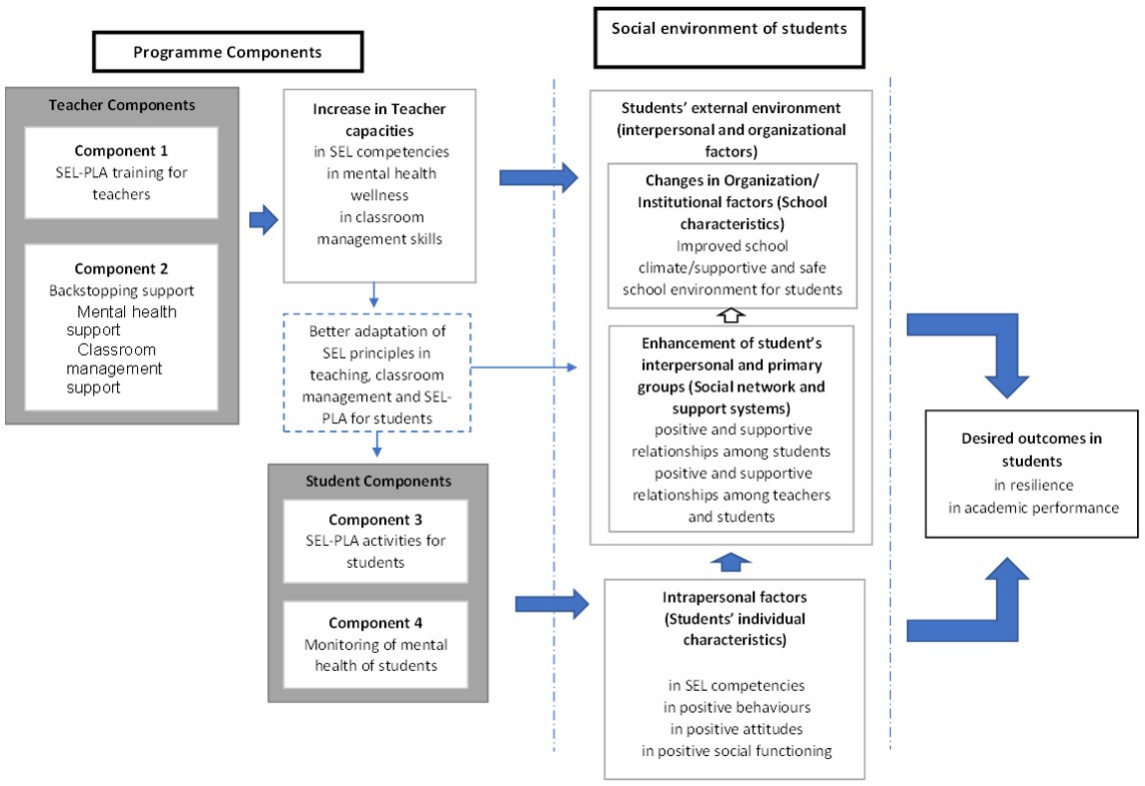
Conceptual framework of the programme.

Fig 2 illustrates the conceptual framework of this programme. The intervention components of the programme target the individual and social environmental factors of the students to improve resilience and academic performance in students. Three levels of the student’s personal and social environment are defined:

1. Intrapersonal level: the first level represents the student’s own individual characteristics. The individual factors targeted by the programme are the student’s SEL competencies, behaviours and attitudes.
2. Interpersonal level: the second level comprises relationships, culture, and society with whom the individual interacts. In the context of this programme, this level refers to factors in the student’s social network and primary support system in the school. In this level the programme targets change to the student’s networks and relationships with fellow students and teachers.
3. Organisational level: the third level refers to factors influenced by the organisational system, in this context–the characteristics of the school. The programme targets change in attitudes and knowledge of teachers which will then translate into changes to the school characteristics, to result in a safe and supportive school climate, conducive for students’ growth and development.

Further, the SEL programme has been conceptualized with specific attention in recognition of the centrality of the refugee communities to the programme in identifying and addressing issues of concern, undertaking critical reflection and action on those issues, and working to resolve the collectively prioritized problems for action. Hence, the design of the programme included a participatory action-research (PAR) approach [27]. PAR is an approach to research in communities that emphasizes participation and action seeking to understand the world by trying to change it, collaboratively and following reflection. It involves researchers and participants working together to understand a problematic situation and changing it for the better. A key aspect of PAR is the importance of the *processes* of knowledge production beyond the mere ‘documentation of needs and perspectives’ [28]. In following the tradition of other action-research projects with refugee youth, the intervention team designing the SEL programme collaborate with refugee stakeholders from the CLC to identify and address issues within the community [29,30] within the programme itself. In this way, the programme seeks to facilitate an empowering approach through processes that strengthen the community’s participation. At the same time, it attempts to be mindful of the differences related to gender, class, educatopn, documentation status, and culture that would have an important bearing on the project.

The first component of the programme (SEL-PLA training for teachers) is meant to increase the capacity of teachers in SEL competencies in teachers and provide strategies for teachers to manage classrooms using SEL principles. The second component, backstopping support, complements the SEL-PLA training for teachers in providing mental health support and reinforcement of further classroom management skills. Teachers will then be able to better adapt SEL principles in the management of their students and in implementing SEL-PLA for their students (Component 3). Enhanced capacity of teachers in SEL principles and class management will then lead to changes in interpersonal and organizational factors. Teachers’ increased SEL understanding, and practices will also be reflected in their attitudes and behaviours with fellow teachers and students, leading to a more positive and supportive school environment. Also, building teachers’ capacity in SEL will contribute to teachers with good SEL competencies who can influence the learning context and the infusion of SEL practices in the classroom. This in turn contributes to the promotion of positive relationships with their students and the promotion of positive relationships among students in their class, simultaneously enhancing students’ social network and support system in school.

The third component, the SEL-PLA with students is targeted at impacting two levels; the student’s characteristics (attitudes, behaviour, social functioning) and the students’ social network and support systems (their teacher and peer network and support in school). The SEL-PLA sessions are meant to increase students’ SEL competencies that will in turn improve student SEL competencies which will be evident in their attitudes, behaviour and social functioning. Improvement in students’ individual characteristics will also interact with interpersonal factors, in the improvement of relationships that will strengthen students’ social network and support systems within the school environment. The fourth component, surveillance of mental health of the students, complements SEL-PLA with students. Mental health issues are known risk factors and moderators of academic performance and resilience [31–33], and may affect the uptake of SEL instruction. These variables are monitored to understand their effects on the resilience building of students.

### 2.4 Study design

A pre-post single arm design combined with qualitative and quantitative methods will be used to address the research questions (see Table 1).

**Table 1:** Methods, data collection instruments and data source according to research questions.

Research Question	Data collection Instruments	Data Source
1. Is PARSEL feasible in the context of CLC in terms of acceptability and implementation, from the perspective of teachers? a. Acceptability b. Implementationi. i. Fidelity ii. Changing needs and support throughout the programme	Focus group discussion (FGD) In depth Interviews Lesson observations Teacher self-report log Focus Group Discussion (FGD) Focus group discussion (FGD) WhatsApp group Reflective open-ended questions and journaling SEL Needs assessment survey Safe, Inclusive, and Respectful Climate survey (School Climate Survey) [39]	Teachers Teachers Programme team Teacher Teacher Teacher Teacher Teacher Teacher Teacher
2. What is the impact of the programme on: a. Student resilience and academic performance b. Student SEL competencies c. Student mental health status d. Teacher mental health status e. Teacher SEL competencies	Child Resilience Questionnaire Student academic grades Social Skills Improvement System Rating Scale (SSIS) [38] Focus group discussion (FGD) SDQ [36] Depression, Anxiety and Stress Scale, 21 items (DASS-21) [37] Pre post tests In depth interviews	Students, Teachers, Parents Teachers Students, Teachers, Parents Teachers Students, teachers, parents Teachers Teachers Teachers
3. Do any of the proposed mechanisms in the conceptual framework explain the link between the programme, resilience and academic achievement? Was the theory of change realised in the study design?	Safe, Inclusive, and Respectful Climate survey (School Climate Survey) [39] Focus group discussion (FGD) Child Resilience Questionnaire Student academic grades Social Skills Improvement System Rating Scale (SSIS) [38]	Teacher Teacher Student, Parent, Teacher Teacher Student, Parent, Teacher

### 2.5 Intervention

The intervention consists of four main components; 1. the training and support for teachers to implement SEL themed participatory learning activities (PLA) for students, 2. backstopping support for teachers 3. the PLA for the students (implemented by the teachers) and 4. mental health surveillance for students.

#### Component 1: The training and support for teachers to implement SEL themed participatory learning activities (PLA) for students and SEL-based classroom management strategies

The first component, training workshops for teachers, involves the teachers being trained in SEL competencies and how to execute PLA for their students, translating their learning in relation to child development, communications with children, and positive discipline into praxis. There are two strategies by which the teachers can impart SEL skills to their students. The first is though SEL based classroom management strategies and the second through SEL themed interactive workshops (or PLA). There are 17 PLA training workshops focussing on working with assets that children bring in spite of the deficits in the environment as well as working proactively through strategies that identify, and prevent and manage psychosocial risks and their impact. (See Appendix 1 for training topics).

#### Component 2: Backstopping support for teachers

The teachers from the CLC are not formally trained as teachers and lack class management skills, requiring more support compared to schools in other settings. Being of refugee background themselves, some teachers have experienced trauma that may have effects on their mental health. In addition, psychological wellness of teachers are known to have bearings on the implementation of SEL curricula, even in developed settings [24]. Therefore, as part of backstopping support, further coaching support to teachers in implementing what they learn from the training sessions will be facilitated. The teachers’ mental health will also be monitored during the programme. Based on emerging data and issues, the project will also facilitate other support which may include counselling, and therapy. This is in recognition of the unique needs of the school teachers in the learning centre. All teachers in the learning centre are refugees themselves and therefore may face some limitations in terms of resources and additional stressors. Further support in the form of feedback sessions and a dedicated WhatsApp chatgroup platform will be provided to teachers throughout the programme.

#### Component 3: The SEL-PLA for the students (implemented by the teachers)

The third component, PLA for the students involves teachers’ implementation of PLA to their students’ 15 interactive workshops. These workshops within a child-focused, age-appropriate framework are meant to facilitate and help students be aware, absorb, reflect and practice SEL principles. These sessions are planned to begin after at least 7 months of teacher training sessions.

#### Component 4: Mental health surveillance for students

The fourth component involves mental health surveillance for the students in the programme. The mental health of the students will be assessed at several timepoints of the programme.

#### Integration of participatory action research (PAR) into the implementation of the Social and Emotional Learning programme

Existing evidence on SEL and the development of resilience and academic achievement in schools are largely from developed countries. SEL is well established within some school systems in developed countries. Refugee schools in developing countries bring along their own challenges such as poor resource settings in terms of material (facilities and learning materials) and human resources (limited training for teachers). Therefore, implementing an SEL based intervention in such settings needs careful assessment, participation and support from all stakeholders involved, especially from the school community.

Participatory action research (PAR) is characterized by finding practical solutions to a problem with the participation of stakeholders through a series of action and reflection [34]. Stakeholders are given a sense of ownership and commitment to the actions implemented, as they themselves are agents of change [35]. PAR stemmed from action research which has been used in education research in various degrees. The participatory nature of PAR allows the teachers to be involved as innovators and concurrently implementers of action [36]. The reflective process involved in PAR enables teachers to develop a sense of ownership and responsibility towards the knowledge they produce, which then requires them to facilitate real changes in classrooms and in education [36]. The thrust of this SEL intervention is in the building and strengthening of teachers’ capacity to deliver and implement the SEL activities and principles of SEL in school. Given that the context of which the programme is intended for is not typical of previous school-based SEL interventions and in recognition of teachers’ role as agents of change in this process, participatory action research (PAR) is a suitable approach to be integrated into the SEL intervention.

**Fig 3:**
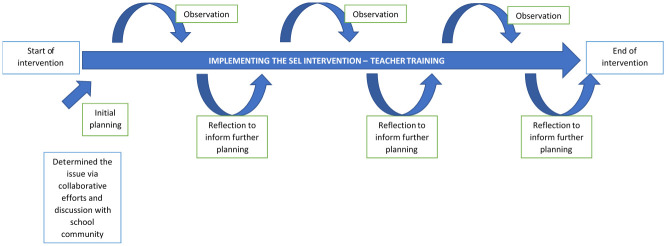
Integration of PAR into the SEL intervention–the iterative reflective inputs and feedback from teachers during their training sessions will be integrated into the planning of subsequent training sessions throughout the intervention period.

**Fig 4:**
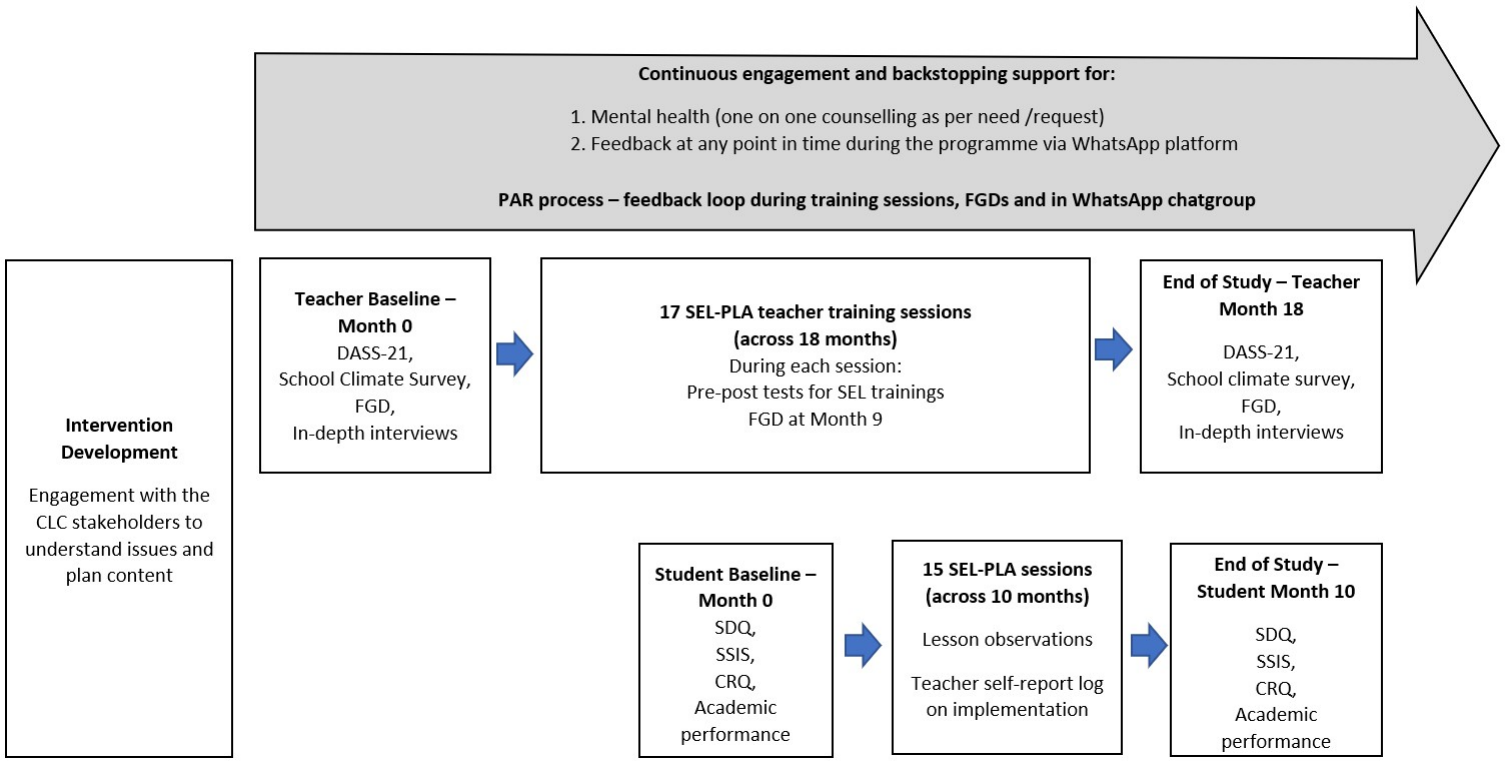
Project flow and assessment schedule for research data collection.

The premise of this SEL intervention and its content had inputs from a series of discussions held with the school leadership prior to its original planning. During the implementation of the intervention, feedback and reflections will be obtained from a series of discussions and reflective activities built into the teacher training sessions. PAR is essentially applied research [37], characterised by a cyclical mode of planning, planning, acting, observing and reflecting [38]. Fig 3 shows how PAR is integrated into the SEL intervention, focusing on teacher trainings. The reflections from teachers based on their knowledge and experience on what would be beneficial for their students and for the successful implementation of the intervention will be used to modify and improve the content of the subsequent training sessions. Focus group discussions will also be held at intervals (targeted at three intervals throughout the intervention–see Fig 4 Project flow) for reflections and possible solutions pertaining the implementation of SEL suited to the school community context. This mutual feedback loop will foster better understanding and buy-in from teachers on the importance of SEL and better understanding on how to enhance the implementation of the intervention to maximise the efficacy.

#### Impact of COVID-19

The SEL-PLA training for teachers and the SEL-PLA with the students were initially planned to be implemented as in-person classroom sessions. Due to COVID-19, lockdowns were imposed and the CLC was forced to move some of their lessons online. The SEL-PLA sessions (both teacher training and student sessions) now incorporate adaptations that can be implemented online. Materials are digitally sent and accessible online. Lessons are redesigned in bite-sized sessions that incorporate interactive and participatory elements to engage the teachers. These resources will be uploaded on a Learning Management System. Although the resources can be accessed asynchronously, the session itself will be run synchronously. Zoom provides common platform for the synchronous sessions. The measurements needed for the evaluation too will shift to use more digital devices, with minimal physical contact.

### 2.6 Participants

#### 2.6.1 Sample

**Participants are from a community learning centre (CLC).** The CLC involved in this programme is located in urban settings in Kuala Lumpur, the capital city of Malaysia. Established in March 2007, the centre was set up to provide education for the children from Myanmar refugee families. Classes ranged from pre-school to secondary school. In the year 2021, there were 16 teachers and 215 students enrolled for the academic year.

#### 2.6.2 The teachers

All teachers from the school (at current baseline, n = 16) will be invited to participate. Teachers will be enrolled into the study if they are willing to provide informed consent and agreeable to commit for the full duration of the programme.

#### 2.6.3 The students and parents

All students aged 6 to 19 still enrolled in the academic year of 2022 will be invited to participate. Students who are willing to provide assent and whose parents or caregivers are willing to provide informed consent will be enrolled into the evaluation. We are aware that there may only be a small number of students that will be part of the collected data at this early stage and also the limited timeframe to observe the effects of the intervention. We intend to plan a full-scale randomised trial once we have as a follow up to this study; hence, there were no power calculations of sample size at this stage. Nevertheless, for a study with an effect size of 0.3 (as per studies in the literature) [15] and a power of 80%, a sample size of at least 90 students would be needed to test the association at 5% levels using two tailed test.

### 2.7 Data collection, measures, outcomes

A mixed methods approach allows a deeper as well as a more comprehensive analysis [39] of this multipronged complex intervention. A concurrent mixed-method design [40] will be employed for this evaluation. Qualitative and quantitative data will be collected concurrently and triangulated.

#### 2.7.1 Primary outcomes and assessment measurement

Feasibility assessment.

Acceptability

In depth interviews and focus group discussions will be conducted with the teachers to assess their perceptions and thoughts about the intervention and its implementation at scheduled timepoints (see Fig 4), specifically focusing on:

1. What are the barriers and facilitators to implementing the activities with their students?
2. Do the teachers see value in the programme as it was delivered?
3. What is the proportion of eligible students who participated in the SEL-PLA sessions with their teachers? (we define an acceptable range as 70% and above)

Implementation

The implementation and process evaluation will be performed during the entire duration of the SEL programme. During the implementation of the structured SEL-PLA activities for students, a trained observer from the programme team will be attending the sessions. The observer will complete a Lesson Observation form collecting information on the following:

- adherence to the intended activities
- the quality of the delivery
- teacher and student engagement during the activities
- duration of the activities (dosage)
- any adaptations performed

Teachers will also be asked to complete self-report logs on the extent of their implementation with the students documenting the following details:

- adherence to the intended activities
- student attendance and participation levels during the activities
- duration of the activities (dosage)
- any adaptations performed

Changing needs, resources and support requested by teachers.

SEL needs assessment and contextual factors will be collected via the SEL needs assessment survey from School Connect [41], the Safe, Inclusive, and Respectful Climate survey [42] and focus group discussions with teachers. The SEL needs assessment survey is designed to be a pre-intervention assessment of overall school climate and students’ SEL skills scale and the Safe, Inclusive and Respectful Climate survey measures the degree to which teachers perceive their school’s environment to be safe and inclusive for their students. SEL needs of the teachers will also be monitored using a WhatsApp chatgroup platform established for this project. Reflective open-ended questions integrated into training sessions will also be used to document and support teacher’s needs.

Our definition of successful implementation would focus on the adherence to the intended activities that were implemented (the fidelity), of at least 70% and dosage executed (at least 70% of the total planned duration).

#### 2.7.2 Secondary outcomes (Effects)

*Student resilience*, *academic performance*, *SEL competencies*, *mental health status*. Resilience will be measured using the Child Resilience Questionnaire, a multi-domain measure of resilience developed by The Childhood Resilience Study [43]. This scale measures five domains of individual (self) and environmental (school and friends, family, and culture) components associated with resilience.

Academic achievement will be assessed using the Academic Performance subsection scores from the teacher report of the Social Skills Improvement System Rating Scales (SSIS) [44]. SSIS is a scale that measures Social Skills, Problem Behaviours and Academic Competence. The average grade point of each student will also be obtained from their school records.

SEL competencies will be measured using pre/post-tests to be administered after SEL-PLA activities are implemented, supplemented by the SSIS [44] subsections in Social Skills, collected from students, teachers and parents.

Mental health of the students will be assessed using the Strengths and Difficulties Questionnaire [45,46], which is a screening tool for psychological adjustment in children and aims to detect emotional or behavioural problems. Data will be collected from teachers, students and parents.

In addition, focus group discussions with teachers will also be used to elicit observed differences in their students before, during and after the implementation of the SEL-PLA activities. Given the limited time teachers have to implement the student SEL-PLA (due to COVID-19 restrictions and lockdowns), quantitative effects may not be immediately measurable. Hence, qualitative observations of the effects of the SEL activities will be elicited from focus group discussions with the teachers as well.

*Teacher mental health status and SEL competencies*. Mental Health will be measured using the Depression Anxiety and Stress Scale, 21-item version (DASS-21) [47]. DASS-21 has three subscales that measures depression, anxiety and stress symptoms in an individual.

Teaching competencies for SEL will be measured using pre-post assessments to be administered at every teacher training session. Focus group discussions with teachers will held at intervals of the study to elicit on their perception of their own SEL competencies, areas to improve on and their confidence in providing a supportive SEL environment for their students.

*Other covariates and factors for the testing of theory*. Other covariates and factors like the changes in the school climate and changes in relationships and social support network of the student will also be monitored. The Safe, Inclusive, and Respectful Climate survey [42] will be used to monitor teachers’ perceived changes in the school environment at the beginning, middle and end of the study. Changes in relationships and social support network will be elicited from periodic focus group discussions with teachers and documented qualitatively.

The relationship between SEL competencies, resilience and academic performance will also be tested.

#### 2.7.3 Assessment schedule

The assessment schedule for research data collection activities is shown in Fig 4.

### 2.8 Data analysis

#### 2.8.1 Quantitative data

For all quantitative data, descriptive statistics will be run with continuous variables summarized using means and standard deviations and categorical variables summarized using frequencies and proportions.

The assessment of intervention implementation will be using the summary measures collected from observations and self-reports.

For the assessment of impact, we will analyse scores from the respective instruments to estimate if there are differences before and after the intervention using T-test, Wilcoxon rank sum test, one-way repeated measures ANOVA or the Friedman Test, depending on whether the data obtained fulfilled the assumptions required for the different type of analyses.

Further, path analysis will be used to test for mediators and moderators of SEL competencies and the relationship with resilience and academic performance in students.

#### 2.8.2 Qualitative data

Qualitative data from reflective exercises, feedback, interviews and focus group discussions will be transcribed and translated to produce transcripts for thematic analysis [48]. Two researchers will independently assign codes and discrepancies in coding will be discussed. Themes with sub-themes will be identified and defined with quotes from the transcripts. The coding of themes will be refined to identify broad overarching issues, linked to the main research objectives.

#### 2.8.3 Triangulation

This protocol incorporates a few approaches to data triangulation [49] to increase the trustworthiness [50] of the analysis. Overall, we will implement methodological triangulation, which is the use of more than one data collection technique (reflections, feedback, interviews, focus group discussion, questionnaires and academic scores). From a data perspective, we will triangulate data from multiple sources (parents, teachers and students). Finally, we will implement researcher triangulation in the qualitative interviews by using at least two analysts in the coding and thematic analysis.

## 3. Discussion

This is a mixed-method evaluation protocol to assess the feasibility and effect of a school-based Social and Emotional Learning (SEL) programme in a refugee learning centre situated in a developing country. The focus of the feasibility will be on the acceptability and implementation [51] of the intervention. This evaluation uses a social ecological framework and incorporates participatory action research approaches. The crux of the study centres on the capacity building and continuous supportive environment for teachers of refugees who are refugees themselves. The formative evidence from this study will be used to plan SEL interventions to be expanded to other refugee learning centres. Data will also be used to design a cluster randomized trial for similar settings to generate evidence to support the efficacy of SEL on the development of resilience and academic achievement.

Our study will contribute to the literature on the educational needs and capacities of refugee learning centres, their educators and of refugee children situated in a country that does not recognise the rights of refugees. More importantly, the nature of the mixed methods and participatory study design allows a more collaborative input from the population of interest, prioritising their role an agent of change within their unique circumstances.
